# Supplementary material for: A randomized controlled trial of N-acetylcysteine in the treatment of early-onset preeclampsia: Study Protocol
Source: PLoS One. 2026 Jun 17;21(6):e0347695. doi: 10.1371/journal.pone.0347695 (PMC13274880; doi:10.1371/journal.pone.0347695)
Supplement: Attachment — Submitted filename: Authors Reply_v6.0.docx [file pone.0347695.s002.docx]

**A Randomized Controlled Trial of N-Acetylcysteine in the Treatment of Early-Onset Preeclampsia: Study Protocol (PONE-D-26-16755)**

**Reply to Editorial Comments**

Thank you for your response to our query, and for addressing all previous comments. We appreciate the efforts to preserve blinding despite the challenges introduced by the documented flavor/smell of NAC. Given this reality, performing a blinding assessment as part of your study would increase confidence in the results. If that is not possible, this should be acknowledged as a limitation. Could you please provide your thoughts on the above and modify the manuscript as necessary?

Reply: We agree that the characteristic sulfur-like odour and taste of NAC pose potential challenges to maintaining effective blinding. In our study design, we are implementing a standardized administration approach in which both NAC and placebo tablets will be taken with Coca-Cola Zero Sugar to mask the potential sensory differences and minimize the risk of unblinding. In addition, we have now included a formal blinding assessment in the Methods section. Specifically, at the end of the intervention period, participants and study personnel will be asked to indicate their perceived treatment allocation (NAC, placebo, or unsure), and the success of blinding will be evaluated by comparing the distribution of these responses between treatment arms (see in red ink, page 11, first paragraph and page 17, first paragraph).

Thank you.

**Prof K.S. Okunade (For the Authors)**
